# Comprehensive measurement of stroke gait characteristics with a single accelerometer in the laboratory and community: a feasibility, validity and reliability study

**DOI:** 10.1186/s12984-017-0341-z

**Published:** 2017-12-29

**Authors:** Sarah A. Moore, Aodhan Hickey, Sue Lord, Silvia Del Din, Alan Godfrey, Lynn Rochester

**Affiliations:** 1 Institute of Neuroscience, Newcastle University Institute for Ageing, Clinical Ageing Research Unit, Campus for Ageing and Vitality, Newcastle University, Newcastle upon Tyne, NE4 5PL UK; 2 Department of Computer and Information Science, Northumbria University, Newcastle upon Tyne, NE2 1XE UK; 3 Auckland University of Technology, Auckland, New Zealand; 4 Institute of Neuroscience (Stroke Research Group), Newcastle University, 3-4 Claremont Terrace, Newcastle upon Tyne, NE2 4AE UK

**Keywords:** Stroke, Gait, Physical activity, Reliability, Validity, Accelerometer

## Abstract

**Background:**

Application of objective measurement of stroke gait with accelerometer-based wearable technology and associated algorithms is increasing, despite reports questioning the accuracy of this technique in quantifying specific stroke-related gait impairments. The aim of this study is to determine the feasibility, validity and reliability of a low-cost open-source system incorporating algorithms and a single tri-axial accelerometer-based wearable to quantify gait characteristics in the laboratory and community post-stroke.

**Methods:**

Twenty-five participants with stroke wore the wearable (AX3, Axivity) on the lower back during a laboratory 2 minute continuous walk (preferred pace) on two occasions a week apart and continuously in the community for two consecutive 7 day periods. Video, instrumented walkway (GaitRite) and an OPAL accelerometer-based wearable were used as laboratory references.

**Results:**

Feasibility of the proposed system was good. The system was valid for measuring step count (ICC 0.899). Inherent differences in gait quantification between algorithm and GaitRite resulted in difficulties comparing agreement between the different systems. Agreement was moderate-excellent (ICC 0.503–0.936) for mean and variability gait characteristics vs. OPAL. Agreement was moderate-poor between the system and OPAL for asymmetry characteristics. Moderate-excellent reliability (ICC 0.534–0.857) was demonstrated for 11/14 laboratory measured gait characteristics. Community test-retest reliability was good-excellent (ICC 0.867–0.983) for all except one (ICC 0.699) of the 19 gait characteristics.

**Conclusion:**

The proposed system is a low-cost, reliable tool for quantifying gait post-stroke with multiple potential applications. Further refinement to optimise gait quantification algorithms for certain gait characteristics including gait asymmetry is required.

## Background

Gait impairments such as reduced gait speed and temporal asymmetry are common after stroke, limiting community ambulation [1] and physical activity [2]. Reduced physical activity predisposes this at-risk population to increased morbidity, stroke recurrence, and further cardiometabolic disease [3].

Quantification of gait after stroke via examination of spatio-temporal characteristics (e.g. step length and velocity, which, in relation to gait outcomes (see below), we term ‘micro’ characteristics [4]) commonly occurs in a gait laboratory. Laboratory testing relies on expensive equipment and technical support, limiting data capture to specialist centres and analysis to a sparse number of gait cycles that are produced under highly controlled conditions. In clinical practice when laboratory facilities are not available, functional measures such as the ten metre walk test [5] and the Dynamic Gait Index [6] are commonly used to measure gait after stroke [5]. These approaches are limited however for three key reasons: measures of gait velocity and function may not detect gait quality or asymmetry; one-off testing in a controlled environment does not inform about day to day variability in performance or the challenges of real-world walking and visual observation and stop watch timing can be limited by observer error. Instrumented walkways such as the GaitRite system are another potential method of measuring gait via a pressure sensitive mat that detects footfall location and timing during walking [7]. Electronic measurement with the GaitRite system reduces observer bias and the system has been shown to have good to excellent intra and inter-rater reliability in sub-acute stroke [8]. One-off testing with the GaitRite system however prohibits understanding of community walking.

The use of accelerometer-based wearable technology (wearables) to quantify micro gait characteristics has gained popularity in stroke research with open-source, data processing platforms facilitating ease of application [9, 10]. Wearable technology offers a low-cost method of quantifying gait in the clinic and can reduce the bias of manual recording techniques [11]. In the community wearable technology can be used to increase ecological validity and reduce observer bias and attentional compensation associated with clinic testing [12].

Capturing levels of physical activity is also possible using wearables [13] and this field appears more advanced in stroke research than measurement of spatio-temporal features of gait which is, by comparison, novel. Traditionally, physical activity outcomes that describe the ‘volume’ of activity after stroke such as step count are reported [14] but these have now been extended to include more nuanced and informative outcomes such as the pattern and variability of activity [15, 16]. We refer to this grouping of volume, pattern and variability physical activity measures as ‘macro’ gait outcomes [4].

Research examining the application of wearables to measure micro and macro gait outcomes in stroke is emerging [17–21]. Although this application of wearable technology is promising, a number of potential problems require consideration for future use of wearable technology in stroke. Potential problems include: application of inertial measurement units with short battery lives necessitating regular recharging (limiting community application) [22]; use of multiple wearables resulting in a high researcher/participant burden [4]; use of engineering terminology to describe gait measures reducing clinical application [4] and validation against manual recording techniques leading to potential bias [21]. The hemiparetic gait pattern adds a further challenge to development of accurate algorithms for detection and processing of discrete spatio-temporal features such as step asymmetry [17]. Also, to date research has focused on measuring either physical activity *or* spatio-temporal aspects of gait, rather than integrating both which would allow for more targeted rehabilitation approaches. The use of a single wearable that could capture both spatio-temporal and physical activity aspects of gait over time in the community could address some of the problems highlighted above. Previous work has demonstrated the feasibility of instrumenting gait with a single wearable positioned on the fifth lumbar vertebrae [23, 24]. This location enables the optimal functionality of the algorithms selected for both spatio-temporal and physical activity aspects of gait in healthy participants. This functionality however, has not been confirmed in stroke where pathological gait is commonly observed.

The aims of this study were to determine the feasibility, reliability and validity of a low-cost open-source system, incorporating algorithms and a single tri-axial accelerometer-based wearable (AX3), to comprehensively quantify gait in terms of both spatio-temporal (micro) and physical activity (macro) outcomes in the laboratory and community post-stroke.

## Methods

Study design: Cross-sectional (Time point 1 and Time point 2) and observational/longitudinal (Week 1 and Week 2).

Setting: Gait laboratory, Clinical Ageing Research Unit, Campus for Ageing and Vitality, Newcastle upon Tyne and community settings in North East England.

### Participants: Inclusion criteria

Community dwelling stroke survivor; at least 1 month post-stroke onset; mild to moderate gait deficit defined by clinical observation of asymmetry of gait including reduced stance time, increased swing time in the affected limb and/or reduced gait speed/balance problems; no changes in gait-related ability over the past month based on self-report and able to walk 10 m with/without a stick (cane or quadropod cane). Exclusion criteria: medical problems other than stroke impacting on gait e.g. osteoarthritis. Participants were recruited via advertisement or therapist referral. All eligible participants were consecutively invited to participate in the study. The study was approved by the Greater Manchester West Research and Ethics Committee. All subjects gave informed written consent for the study according to the Declaration of Helsinki.

### Demographic and clinical measures

Participant age, gender, date of stroke, stroke type (Oxford Community Stroke Project Classification [25]), stroke impairment (National Institute of Health Stroke Scale [26] hemiplegia (clinical observation by two independent experienced clinicians), walking stick use, ankle foot orthosis (AFO) use, height and weight were recorded at baseline.

### Test protocol for laboratory-based outcomes

Participants were asked to walk for 2 minutes continuously around a 25 m track at self-selected speed in a laboratory instrumented with a GaitRite system whilst wearing an AX3 wearable (see instrumentation) affixed with double sided tape and Hypafix (BSN Medical Limited, Hull, UK) at the fifth lumbar vertebrae (L5). A continuous rather than intermittent walk was chosen based on previous methodological findings [27].

### Validity

**Fig. 1:**
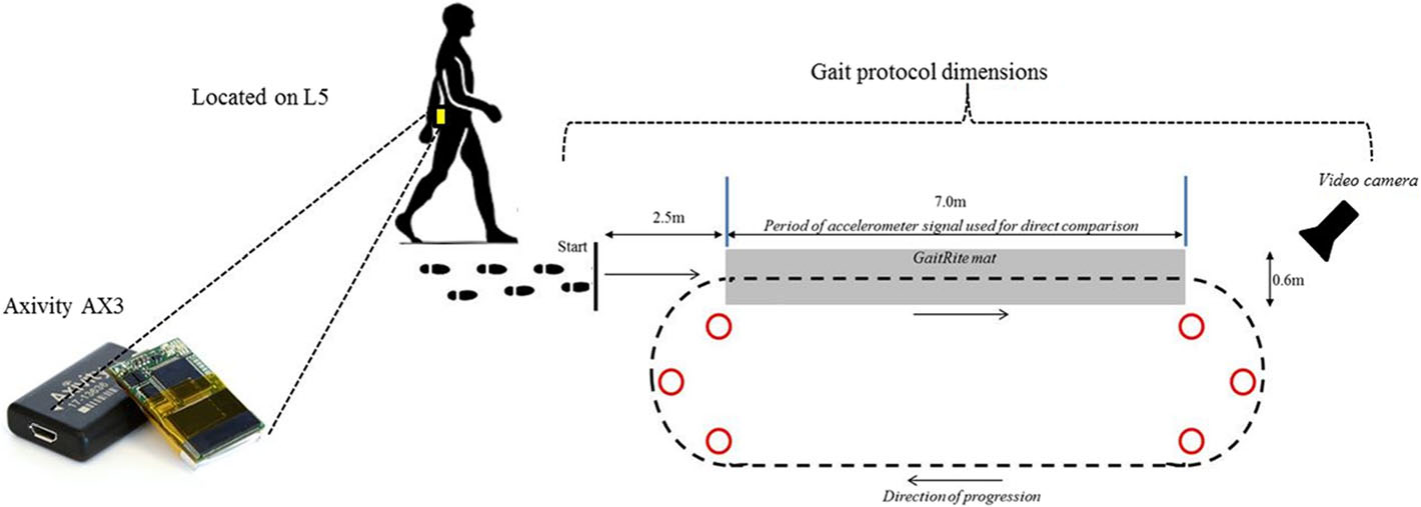
Clinic protocol and positioning of AX3 wearable

Three tools were used during the walking trial to establish the validity of the AX3 wearable: GaitRite instrumented walkway; high grade wearable data capture system (OPAL, APDM) and video (to facilitate manual step count checking and visual inspection of stroke related impairment). The GaitRite mat was placed in the 25 m circuit (see instrumentation and Fig. 1) to allow measurement of the spatio-temporal aspects of gait. The single wireless OPAL device was affixed directly adjacent to the AX3 at L5 and held in place by double sided tape and Hypafix (see instrumentation).

#### Test-retest reliability

Test-retest reliability of the AX3 was established by repeating the walking trial 1 week later (± 2 days) and comparing measures taken at the first walking trial (T1) with the second (T2). Walking aid and AFO conditions were matched over trials.

### Test protocol for community-based outcomes

In order to assess feasibility and test-retest reliability of the AX3 wearable in the community participants were asked to wear the AX3 wearable for two consecutive 7 day periods. After completing the laboratory protocol at T1 participants were provided with an AX3 wearable to wear for 7 days in the community (Week 1). The wearable was worn continuously except for skin cleaning or during bathing/swimming (the wearable was water resistant but not waterproof). Participants, or a family member who could assist, were instructed how to remove and reattach the wearable. The data from the wearable was downloaded on the participants return to the clinic at T2. The participants were then asked to wear the wearable for a second consecutive 7 days (Week 2). The wearable was returned by participants in a pre-paid envelope and data downloaded. During the 2 week measurement period participants completed a daily log of daily activities wear time and any problems with the wearable. This log was reviewed at T2 clinic appointment and each participant was questioned on comfort and application of the wearable. This data was used alongside wearable data capture to assess feasibility.

### Instrumentation

#### Low-cost wearable

The AX3 is a single tri-axial accelerometer-based wearable (AX3, Axivity, York, UK https://axivity.com/, cost ≈£100, dimensions 23.0 mm × 32.5 mm × 7.6 mm). The AX3 weighs 11 g, has a memory of 512 Mb and a battery life of 14 days. AX3 data capture is 100 Hz (16-bit resolution) at a range of ±8 g. Recorded AX3 accelerations were stored locally on the device’s internal memory and downloaded upon the completion of each walking trial.

#### Video

A Samsung W200, 25 Hz video camera was used to record walking trials to allow visual inspection for manual counting of steps within the gait task and visual observation of asymmetry.

#### Instrumented walkway

Dimensions were 7.0 m × 0.6 m and had a spatial accuracy of 1.27 cm and a temporal accuracy of one sample (240 Hz, ~4.17 ms) (GaitRite: Platinum model GaitRite, software version 4.5, CIR systems, NJ, USA). Gaitrite has been used extensively in the quantification of spatio-temporal measures in stroke [8, 28].

High-cost wearable system The OPAL wireless accelerometer-based wearable system (APDM, Inc., Portland, OR, USA, https://www.apdm.com/wearable-sensors) comprises a tri-axial accelerometer, gyroscope and magnetometer [18] (128 Hz, 8Gb, 16 h of logging 8 h of streaming). The OPAL system has been used previously in stroke for quantification of spatio-temporal measures [18, 20]. Only accelerometer data were used in the analysis.

#### Data synchronisation

Video, GaitRite and OPAL were synchronised in time with the AX3 recordings. The quartz-stabilized real-time clock of the AX3 and OPAL systems (accuracy: 20 ppm) were synchronized with the computer used for the GaitRite recordings, and for each walking trial, the start and stop time were recorded by the assessor. Start and stop times were subsequently input to a bespoke MATLAB program that automatically segmented and analysed the accelerometer data. AX3 and OPAL data corresponding to steps leading to and after contact with the GaitRite were excluded from analysis so a direct comparison could be made between the wearables and instrumented walkway.

### Wearable algorithms

#### Micro (spatio-temporal) outcomes

The 14 temporal and spatial gait characteristics defined by Lord et al. (2013) [29] were quantified from the AX3 and OPAL wearables and GaitRite. Mean gait values were calculated for step time, stance time, (time stance foot was in contact with the ground for a given stride), swing time (duration a foot was not in contact with the ground for a given stride), step length and step velocity. The standard deviation from all steps was calculated to determine step variability. Asymmetry was calculated as the absolute difference between consecutive left and right steps. Algorithm and data segmentation techniques applied to both the AX3 and the OPAL data and rationale have been described in full previously [9]. In brief the vertical acceleration underwent continuous wavelet transformation to estimate initial contact and final contact in the gait cycle while the inverted pendulum model was used in conjunction with those outcomes for spatial data.

#### Macro (physical activity) outcomes

A validated free-living algorithm [30] was used to quantify macro characteristics (e.g. volume, pattern and variability) Volume was quantified as total daily step count. Pattern was quantified as number of daily walking bouts (minimum bout length defined as three steps [31]), mean length of walking bouts (s) and alpha (α) as the distribution of ambulatory bouts (a lower α indicates that the distribution is derived from a greater proportion of longer bouts [32, 33]). Variability was described as the within subject variability of bout length calculated from the maximum likelihood technique due to non-normality of data [34]. A high variability indicates a more varied pattern of walking.

#### Statistical analysis

Analysis was completed using SPSS v23 (IBM). Normality of data was tested with a Shapiro-Wilk test. Descriptive statistics (median and interquartile range) were calculated for gait characteristics measured by AX3 and OPAL wearables and GaitRite. Bland-Altman plots were used to visually check for non-linear or heteroscedastic distributions of error between AX3 and clinic references. Data for two participants who wore a fixed plastic AFO were removed from the analysis as individual data analysis (including video observations) revealed the algorithms applied were not appropriate for these two participants. This finding was likely due to fixed nature of the AFO impacting on heel strike and the performance of the algorithm which was developed based on the detection of initial and final contact within the gait cycle. Individuals who wore a push aequi AFO were able to achieve some degree of plantarflexion therefore were included in the analysis.

Agreement between AX3 measurements and video, OPAL and GaitRite and on AX3 measures at T1 compared with T2 were formally tested using intraclass correlation coefficient, Spearman’s rank correlation coefficients (*r)* and limits of agreement (LoA) expressed as a percentage of the mean of the two measures and the 95% LoA.

For all analysis statistical significance was set at *p* < 0.05. Predefined acceptance ratings for ICCs were set at excellent (≥.900, 0.0–4.9%), good (0.750–0.899, 5.0–9.9%), moderate (0.500–0.749, 10.0–49.9%) and poor (<0.500, >50.0%) [35].

### Participant characteristics

Participant demographic and clinical data are shown in Table 1. The sample had a heterogeneous mix of gait speeds (range 0.14–1.4 m/s). Video observation of participants by two experienced clinicians showed 15 of the 23 participants (65%) had marked hemiplegia (moderate to severe) impacting on gait symmetry.

**Table 1:** Participant characteristics

Demographics (*n* = 25)	
Gender (male/female)	19/4
Age (years)	63 ± 11
Stroke characteristics	
Time since stroke (months)	66 ± 48 (range 5–201)
Stroke subtype (OCSP)	
Total anterior circulation	11
Partial anterior circulation	6
Lacunar	3
Posterior circulation	3
Stroke impairment (number (%))	
NIHSS score (0–40)	4 ± 3 (range 0–11)
NIHSS lower limb score (0–4)	1 ± 0.7 (range 0–3)
Walking speed (m/s)	0. 9 ± 0.4
Marked Hemiplegia (Yes/No)	15/8
Walking aid	3 (13%)
Push Aequi ankle foot orthosis	4 (17%)

*OCSP* Oxford community Stroke Project, *%* Percentage, *NIHSS* National Institute for Health Stroke Scale

## Results

### Laboratory-based measures Feasibility

25/25 participants completed the testing protocol with no adverse events or AX3 wearable missing data. We were able to quantify all fourteen spatio-temporal gait characteristics in all 23 participants included in the analysis.

### Validity (AX3 wearable system vs. clinic references)

Absolute agreement between AX3 wearable and video step count was good (median 210 (interquartile range 41) vs. 206 (36), median difference 4 (2%), ICC 0.899, *P* ≤ 0.01, LoA expressed as a percentage of the mean of the two measures 0.4 (95% LoA 49.6, −38.6).

Table 2 demonstrates agreement for spatio-temporal measurement between AX3 wearable system and two references (GaitRite and OPAL). Absolute agreement between the system and GaitRite was moderate to good for mean step velocity, step time, stance time, step time and stance time variability (ICCs 0.724–0.802) but poor for all other micro characteristics. Absolute agreement between the system and OPAL was moderate to excellent for all mean and variability characteristics and swing and stance time asymmetry (ICCs 0.504–0.974) but poor for step length and step time asymmetry. Overall agreement between the system and references was superior for mean and variability characteristics compared with asymmetry characteristics.

**Table 2:** Laboratory based agreement between AX3 wearable system, Gait Rite and OPAL for micro gait characteristics

Variable	AX3 wearable system vs. GaitRite			AX3 wearable system vs. OPAL		
	ICC	*r*	LoA % (95% LoA)	ICC	*r*	LoA % (95% LoA)
Micro gait characteristics						
Mean						
Step velocity (m/s)	0.744**	0.632**	1.1 (0.06, −0.043)	0.923**	0.871**	0.4 (0.19, 0.22)
Step length (m)	−0.411	0.035	1.5 (0.56, −0.35)	0.831**	0.784**	0.6 (0.22, −0.18)
Step time (s)	0.797**	0.917**	1.1 (0.38, −0.38)	0.890**	0.948**	0.6 (0.25, −0.17)
Swing time (s)	0.431*	0.558**	1.3 (0.40, −0.21)	0.900**	0.944**	0.8 (0.22, −0.17)
Stance time (s)	0.758**	0.902**	1.2 (0.50, −0.60)	0.876**	0.871**	0.6 (0.28, −0.17)
Variability						
Step velocity (m/s)	0.052	0.261	3.8 (0.28, −0.05)	0.658**	0.503*	2.1 (0.17, −0.23)
Step length (m)	0.457**	0.121	2.9 (0.175,-0.036)	0.884**	0.616**	1.6 (0.08, −0.104)
Step time (s)	0.802**	0.688**	4.5 (0.25, −0.13)	0.972**	0.940**	1.6 (0.09, −0.10)
Swing time (s)	0.389	0.694**	6.4 (0.33, −0.15)	0.968**	0.928**	1.7 (0.1, −0.11)
Stance time (s)	0.724**	0.766**	4.3 (0.26, −0.12)	0.974**	0.936**	1.5 (0.09, −0.11)
Asymmetry						
Step length (m)	0.442	0.001	4.5 (0.25, −0.18)	0.464	0.381	3.6 (0.2, −0.22)
Step time (s)	0.178	0.601**	3.9 (0.49, −0.75)	0.492*	0.558**	4 (0.13, −0.08)
Swing time (s)	0.320	0.454*	6.7 (0.23, −0.33)	0.539*	0.733**	4.7 (0.13, −0.08)
Stance time (s)	0.439	0.570**	6 (0.216, −0.30)	0.504*	0.592**	4.9 (0.15, −0.09)

Vs. versus, *ICC* Intraclass correlation coefficient, *r* Spearman’s rank correlation coefficient, *LoA%* limits of agreement as a percentage of the mean of the two systems

**p ≤ 0.01 *p ≤ 0.05

### Reliability

Table 3 demonstrates the agreement between laboratory AX3 wearable system micro outcome measurement (T1 vs. T2). Absolute agreement was moderate to excellent (ICCs 0.534–0.858) for all measures except mean step length, step velocity variability and step length asymmetry. Relative agreement was moderate to excellent (*r* 0.525–0.941) for all measure except step velocity variability and step length asymmetry.

**Table 3:** Laboratory based test-retest reliability (1 week apart) for AX3 wearable system micro gait measurement

Variable	Median (IQR)		Correlations/agreements			
	Time point 1	Time point 2	Median difference (%)	ICC	LoA % (95% LoA)	*r*
Mean						
Step velocity (m/s)	1.08 (0.40)	1.08 (0.345)	0 (0%)	0.534*	1.0 (0.34, −0.30)	0.547**
Step length (m)	0.657 (0.113)	0.642 (0.140)	−0.015 (−2%)	0.419	1.0 (0.31, −0.32)	0.572**
Step time (s)	0.600 (0.130)	0.601 (0.123)	−0.001(0.2%)	0.844**	1.0 (0.28, −0.3)	0.941**
Swing time (s)	0.485 (0.174)	0.467 (0.117)	−0.018 (−4%)	0.858**	1.0 (0.24, −0.28)	0.926**
Stance time (s)	0.743 (0.136)	0.740 (0.130)	−0.003 (−0.4%)	0.819**	0.78 (0.32, −0.34)	0.930**
Variability						
Step velocity (m/s)	0.150 (0.053)	0.162 (0.130)	0.012 (7%)	0.343	3.7 (0.33, −0.26)	0.427*
Step length (m)	0.082 (0.077)	0.123 (0.131)	0.041 (33%)	0.632**	2.8 (0.21, −0.13)	0.693**
Step time (s)	0.056 (0.097)	0.082 (0.208)	0.026 (32%)	0.777**	4.17 (0.36, −0.24)	0.886**
Swing time (s)	0.063 (0.098)	0.082 (0.168)	0.019 (23%)	0.793**	4.1(0.33, −0.23)	0.901**
Stance time (s)	0.062 (0.104)	0.087 (0.195)	0.025 (29%)	0.784**	4 (0.36, −0.24)	0.909**
Asymmetry						
Step length (m)	0.084 (0.216)	0.106 (0.125)	0.022 (21%)	−0.194	5.1 (0.36, −0.32)	0.015
Step time (s)	0.045 (0.085)	0.036 (0.077)	0.009 (25%)	0.813**	3.6 (0.14, −0.12)	0.628**
Swing time (s)	0.044 (0.077)	0.035 (0.082)	−0.009 (26%)	0.857**	3.3 (0.12, −0.10)	0.545**
Stance time (s)	0.045 (0.087)	0.042 (0.075)	−0.003 (7%)	0.857**	3.2 (0.11, −0.11)	0.525**

*T1* Time point 1, *T2* Time point 2, *IQR* Interquartile range, *ICC* Intraclass correlation coefficient, *LoA%* limits of agreement as a percentage of the mean of the two systems, *r* Spearman’s rank correlation coefficient

**p ≤ 0.01 *p ≤ 0.05

### Community based outcomes

#### Feasibility

25/25 participants completed the community free living protocol 24 h a day for 7 days, only removing to bathe. There were no missing data sets. The AX3 battery life lasted for over the seven-day monitoring period. Participant logs and questioning during clinic visits indicated participants found the AX3 monitor comfortable to wear and easy to apply. Participants with upper limb impairment reported they either got an informal carer to assist with the application or managed an adapted technique of application with one arm.

Table 4 demonstrates agreement between community habitual physical activity and spatio-temporal measurement by AX3 wearable system (Week 1 vs. Week 2). Absolute agreement was moderate to excellent for all measures (ICCs 0.668–0.982).

**Table 4:** Community based test-retest reliability for AX3 wearable system micro and micro gait measurement

Variables	Median (IQR)		Agreement			
	Week 1	Week 2	Median difference (%)	ICC	LoA % (95% LoA)	*r*
Macro characteristics						
Total daily step count	7825 (6428)	7191 (5920)	−634 (−9%)	0.917**	0.9 (3138, −4470)	0.892**
Mean walking bout length (s)	17.12 (2.56)	16.3 (6.2)	−0.82 (−5%)	0.919**	0.4 (3.036, −3.565)	0.894**
Total number of daily walking bouts	535 (288)	462 (237)	−73 (16)	0.867**	2 (226,-272)	0.823**
Alpha (unit less)	1.614 (0.535)	1.615 (0.068)	0.001 (0.06)	0.948**	0.1 (0.05, −0.046)	0.853**
Variability (s)	0.840 (0.057)	0.842 (0.105)	0.002 (0.2%)	0.887**	0.2 (0.08, −0.094)	0.801**
Micro characteristics						
Mean						
Step velocity (m/s)	1.05 (0.165)	1.07 (0.152)	0.02 (1.9)	0.958**	0.17 (0.087, −0.093)	0.914**
Step length (m)	0.610 (0.085)	0.610 (0.085)	0 (0)	0.969**	1.2 (0.034, −0.038)	0.940**
Step time (s)	0.613 (0.353)	0.622 (0.38)	0.009 (1)	0.951**	0.07(0.022, −0.018)	0.889**
Swing time (s)	0.474 (0.032)	0.475 (0.030)	0.001 (0.2)	0.962**	0.07 (0.02,-0.013)	0.925**
Stance time (s)	0.764 (0.040)	0.766 (0.047)	0.002 (0.2)	0.942**	0.1 (0.026, −0.026	0.863**
Variability						
Step velocity (m/s)	0.385 (0.048)	0.383 (0.042)	−0.002 (−0.5)	0.905**	0.2 (0.04, −0.0345)	0.914**
Step length (m)	0.162 (0.021)	0.159 (0.017)	0.003 (−2%)	0.944**	0.12 (0.034, −0.0384)	0.940**
Step time (s)	0.183 (0.041)	0.181 (0.044)	−0.002 (−1)	0.982**	0.17 (0.018, −0.0149)	0.889**
Swing time (s)	0.152 (0.040)	0.150 (0.039)	−0.002 (−1.3)	0.983**	0.15 (0.0119,-0.0113)	0.925**
Stance time (s)	0.193 (0.042)	0.193 (0.044)	0 (0)	0.982**	0.2 (0.0189, −0.0169)	0.863**
Asymmetry						
Step length (m)	0.082 (0.019)	0.090 (0.021)	0.008 (9%)	0.668**	0.7 (0.0337, −0.0245)	0.599**
Step time (s)	0.123 (0.037)	0.120 (0.044)	−0.003 (−2.5)	0.875**	0.6 (0.0357, −0.0344)	0.788**
Swing time (s)	0.109 (0.040)	0.101 (0.049)	−0.008 (−8%)	0.925**	0.5 (0.026, −0.0261)	0.850**
Stance time (s)	0.123 (0.04)	0.109 (0.030)	−0.01 (−13%)	0.878**	0.6 (0.0357, −0.0398)	0.742**

*IQR* Interquartile range, *s* seconds, *ICC* Intraclass correlation coefficient, *LoA%* limits of agreement as a percentage of the mean of the two systems, *r* Spearman’s rank correlation coefficient

**p ≤ 0.01 *p ≤ 0.05

## Discussion

This is the first study to investigate the feasibility, reliability and validity of a low-cost system incorporating algorithms and a single tri-axial accelerometer-based wearable (AX3) in quantifying a comprehensive assessment of both micro and macro gait characteristics post-stroke. The feasibility of using the AX3 wearable system was excellent with individuals with mild to moderate gait impairment following stroke. In the laboratory validity of the AX3 wearable system measurement of micro characteristics was assessed by comparison to three references (video, GaitRite, OPAL) with positive results. Test re-test reliability of the AX3 wearable system in both the laboratory and the community was moderate to excellent for all bar three of the gait measurements.

Breaking down the raw signal from a single wearable to quantify discrete gait characteristics is a potentially useful approach for quantifying specific gait impairments post-stroke and allows ‘real world’ measurement of these characteristics. Quantification of spatio-temporal characteristics by the AX3 system was compared with two references (GaitRite and OPAL). Agreement for mean gait characteristics between the AX3 wearable system and GaitRite was moderate to good for three measures, but poor for swing time and step length. The poor agreement for swing time may have been due to a limitation of the algorithm which estimated swing time from the difference between stride time and stance time which could lead to small inaccuracies [9]. Poor agreement between step length may have been due to the algorithm as it is based on a healthy model of gait assuming a rhythmical, linear and compass gait cycle (circular trajectory [36]) which is often not present with marked asymmetry common post-stroke and consequently the model may require refinement in this population. Indeed, a previous study using a single inertial measurement unit has indicated that a higher error rate is present in the detection of initial contact, swing time and stance time in pathological gait (including hemiplegic gait) in comparison to healthy elderly individuals [18]. Previous studies have also indicted it may be necessary to apply an individual correction factor to estimate step/stride length with accelerometry in people with neurological conditions including stroke [21].

Although GaitRite has been used in the stroke population to quantify gait characteristics [28], limitations have been observed when making comparisons of wearable measures to instrumented walkways [9]. Limitations relate to inherent differences in mechanisms of data capture as instrumented walkways such as GaitRite use pressure sensing to capture discrete foot falls whereas wearables continuously track acceleration This may have been the reason that the agreement between AX3 wearable system and GaitRite was poor on a number of measures. Agreement between the two wearable systems was better on mean, variability and asymmetry characteristics compared to agreement between the wearable system and the instrumented walkway.

Gait asymmetry is a common consequence of stroke and target of rehabilitation. Formal statistical tests of agreement indicated poor levels of agreement for asymmetry characteristics between the system and references. To explore these findings further individual participant data and formal agreement testing was conducted to explore if gait asymmetry (defined by video observation by experienced stroke clinicians and NIHSS lower limb scores) affected algorithm accuracy (data not reported). Post-hoc analysis indicated agreement was inferior for those with higher levels of asymmetry indicating this requires further exploration and algorithms may require refinement for those with marked gait asymmetry, or alternative placement and algorithms sought., Placement of a wearable sensor with appropriate adaptation of algorithms on each lower limb rather than the lower back may be more suitable for detecting asymmetry gait characteristics [18] but this would double patient burden, cost and increase technical complexity for synchronisation and data fusion. The reliability and validity of bilateral ankle accelerometers has been previously established in a small convenience sample (*n* = 12) for determining walking speeds and bouts of walking activity in the clinic after stroke [19] but the study did not explore gait asymmetry. More recent work on a small sample of hemiparetic stroke participants (*n* = 10) has indicated promise for accurate measurement of spatio-temporal parameters of gait with a system incorporating magneto-inertial units positioned on both ankles [20]. These results will need to be confirmed in a larger sample and are potentially limited by the use of magneto-inertial units requiring frequent recharging.

Alongside the novel use of wearables to quantify spatio-temporal gait characteristics post-stroke, wearables have been used for some time to capture physical activity outcomes. Review of psychometric data, however, has indicated that no single device is ideal at present for the measurement of physical activity after stroke [13]. As daily step count is linearly associated with reduced long-term all-cause mortality [37], and daily step count can be reduced by stroke impairments, it is imperative we have accurate measures of this variable post-stroke. Step count estimated by the system proposed here was accurate across a range of gait speeds when compared to video.

Establishing test retest reliability is important to determine whether there is consistency of measurement across time and subsequent use for longitudinal measurement of gait. Test-retest reliability was excellent for all macro characteristics captured in the community (ICC 08.67–0.917) and good to excellent for all except one of the 14 ‘micro’ characteristics. A recent summary of the test-retest reliability of physical activity measurement using wearable devices in stroke indicated that test retest reliability varied markedly across devices (ICCs 0.68–0.989) [13]. Findings from a number of studies testing measurement on multiple occasions indicted that the Step Watch Activity Monitor was the most reliable device for use to capture step count after stroke. Excellent test-retest reliability for step count was also demonstrated for the AX3 wearable in the current study and the AX3 also allowed for the reliable measurement of pattern and variability of gait activity and spatio-temporal gait characteristics. The AX3 wearable could be used to measure intervention response across a comprehensive range of gait measures in the laboratory and the community after stroke.

### Limitations

Study limitations include the sample’s relatively high average gait speed and mild level of impairment as measured by the NIHSS meaning the sample may not have been representative of a typical stroke population. The use of gait speed and the NIHSS scale to characterise the sample did not allow the capture of data on quality of gait and hemiplegia. Individual levels of gait asymmetry and hemiplegia were assessed by clinical observation from video and indicated that almost two thirds of the sample had moderate to severe hemiplegia resulting in gait asymmetry. The sample may have been representative of community dwelling stroke survivors, but this data was not captured by the selected measures. This subjective data would have been strengthened by the inclusion of a measure of impairment such as the Fugl-Meyer motor assessment [38] The study excluded participants who wore a fixed ankle foot orthosis (AFO) due to the impact of the AFO on heel strike, meaning algorithms applied were unsuitable. Results from this study can therefore only be applied to stroke survivors who do not use a fixed AFO. The sample was not powered but was based upon previous sample sizes of studies of a similar nature [39, 40]. The study compared AX3 wearable system measures to video, and two other high-cost systems: GaitRite and OPAL. Comparing wearable measures to a camera-based motion capture system would have provided an alternative gold standard that may be worth considering in the future.

## Conclusion

Although the low-cost AX3 wearable system demonstrates promise as a feasible and reliable tool with which to measure a comprehensive range of gait characteristics after stroke further work is needed to establish the system’s validity. Gait asymmetry is a common problem after stroke and the systems current algorithms will need to be refined/developed in order to capture this complex pathological gait characteristic.

Multiple applications in the stroke population including: monitoring symptoms; determining intervention and therapeutic effects; analysing relationships between daily fluctuations in activity and stroke impairments such as fatigue; and for patient feedback and assisting with self-management programmes, highlight the important role wearable systems have in future stroke management and the need for further refinement in this area.

## Abbreviations

AFO: Ankle foot orthosis
Hz: Hertz
ICC: Intraclass correlation coefficient
L5: Fifth lumbar vertebrae
m/s: Meters per second
ms: Milliseconds
NIHSS: National Institute of Health Stroke Scale
T1: Time point 1
T2: Time point 2
